# Musculoskeletal Complaints (Pain and/or Stiffness) and Their Impact on Mortality in the General Population. The Tromsø Study

**DOI:** 10.1371/journal.pone.0164341

**Published:** 2016-10-13

**Authors:** Ole Fredrik Andorsen, Luai Awad Ahmed, Nina Emaus, Elise Klouman

**Affiliations:** 1 Department of Community Medicine, Faculty of Health Sciences, University of Tromsø –The Arctic University of Norway, Tromsø, Norway; 2 Department of Health and Care Sciences, Faculty of Health Sciences, University of Tromsø –The Arctic University of Norway, Tromsø, Norway; 3 Institute of Public Health, College of Medicine and Health Sciences, United Arab Emirates University, Al Ain, United Arab Emirates; Garvan Institute of Medical Research, AUSTRALIA

## Abstract

**Background:**

The long-term consequences of chronic pain and/or stiffness from the musculoskeletal system (musculoskeletal complaints: MSCs) have not been well explored. The aims of this study were to investigate whether MSCs reported at baseline influence all-cause and cause-specific mortality during 21 years follow-up of a general Northern Norwegian adult population.

**Methods:**

A total of 26,977 men and women aged 25–97 years who participated in the 1994–1995 survey of the Tromsø study (response rate 77%) were included in the present prospective cohort study. Baseline data were collected from the 1994–1995 survey and information on death and emigration was taken from the National Register of Norway. Cox regression analyses were performed to examine if MSCs predicted risk of mortality.

**Results:**

5693 (21.1%) participants died during follow-up. Mean time between entry into the survey and death or emigration was 18.6 years (standard deviation 4.87) for all-cause mortality. There was an increased risk of death among those with MSCs at baseline in the crude Cox regression model. However, the multivariable model revealed no significant association between MSCs at baseline and all-cause mortality by sex (women: hazard ratio [HR] = 0.93, 95% confidence interval [CI]: 0.85–1.01; men: HR = 0.93, 95%CI: 0.85–1.01). Furthermore, no significant associations were found between widespread MSCs at baseline and all-cause mortality in multivariable models (women: HR = 0.90, 95%CI: 0.80–1.01; men HR = 0.87, 95%CI: 0.76–1.00). Analyses on cause-specific mortality did not reveal any significant results.

**Conclusion:**

MSCs are not independently associated with increased risk of death from cardiovascular disease, cancer, or death from all causes.

## Background

In the Western world, chronic pain and/or stiffness from the musculoskeletal system (musculoskeletal complaints: MSCs) are highly prevalent in all age groups [1–4], and reports indicate an alarming increase among the younger population [5, 4, 6]. Economic consequences due to MSCs are huge and MSCs are currently receiving more attention in clinical and research settings through initiatives such as the Bone and Joint decade [7]. Nevertheless, about 40% of sick leave and a large proportion of disability pensions in Norway are because of MSCs [8], and the proportion is increasing [9].

Despite individual suffering and early disability, the long-term consequences of MSCs have not been well explored. In a previous cross-sectional study, we reported that the prevalence of severe MSCs peaked around mid-life, in contrast to the prevalence of mild MSCs, which steadily increased with age [3]. One possible explanation for this finding is higher mortality among those reporting severe MSCs. Current knowledge on the relationship between MSCs and hard end-points is sparse and has rendered conflicting results. Some studies have indicated an increased mortality among individuals with chronic MSCs [10–14], with the main causes of death being cardiovascular disease and cancer, whereas other studies have shown no association between MSCs and mortality [15–19]. Both prospective and cross-sectional studies have suggested that several negative health determinants, such as smoking, mental health complaints, low educational level, and physical inactivity, are associated with MSCs [20–23, 3]. Thus, MSCs might share risk factors with known deadly diseases such as cardiovascular diseases and cancer [24, 25], further complicating the relationship between MSCs and mortality.

In a systematic review from 2014, Smith et al concluded that the small number of studies and the heterogeneity between them made it difficult to provide a clear picture of the association between chronic pain and mortality. The authors concluded that further research should focus on how health, lifestyle, and social and psychological factors could influence this relationship in large population-based studies [26].

Since MSCs are highly prevalent in the general population, only a small increase in mortality due to MSCs will have large impact on the total mortality rates in a community. Therefore, using data from a large prospective study of a general Northern Norwegian adult population, we aimed to investigate the association between MSCs reported at baseline and all-cause and cause-specific mortality after up to 21 years of follow-up after adjusting for several potential confounders.

## Methods

### Study design

The Tromsø study is a longitudinal, population-based, multi-purpose health study carried out in the municipality of Tromsø, Northern Norway. The study currently consists of six health surveys (Tromsø 1–6); Tromsø 1 was conducted in 1974 and Tromsø 6 in 2007–2008. Data collection for Tromsø 7 is ongoing and will be complete by the end of 2016. The participation rate for all six surveys has ranged from 65% to 77% [27]. The present analyses used the data from Tromsø 4 as baseline data. Tromsø 4 was carried out in 1994–1995 and is the largest of the six completed surveys. All inhabitants of Tromsø over 25 years of age (n = 37,558) were invited to Tromsø 4, and 26,977 attended (12,865 men and 14,293 women) and were eligible for inclusion in this analysis. Excluding those who had moved out of Tromsø or died prior to attendance gave a participation rate of 77%. All participants of Tromsø 4 completed two questionnaires and attended a medical examination. The first questionnaire was sent by post with the invitation (Q1) and the second one (Q2) was completed at the time of the medical examination.

### Variables

Baseline data on age, sex, smoking status, mental health complaints, educational level, leisure time physical activity, self-reported chronic diseases (CVD, diabetes and asthma) and MSCs were taken from Q1, and data on cancer was taken from Q2. Both of the Tromsø 4 questionnaires are available on the Tromsø Study’s website (https://en.uit.no/prosjekter/prosjekt?p_document_id=80172) [28].

Based on questionnaire information, participants were categorized as current or not current smokers. Mental health complaints were assessed using the dichotomized CONOR mental health index (CONOR-MHI); CONOR-MHI <2.15 indicated no mental health complaints and CONOR-MHI ≥2.15 indicated complaints such as anxiety and/or depression [29]. Educational level was collapsed from five to two levels: <13 years and ≥13 years of education. A four-level physical activity index (sedentary, low, moderate, and high) was computed based on the two questions on amount of light and hard physical activity level per week [30]. Self-reported chronic diseases included cardiovascular disease, diabetes, asthma, and cancer, and participants were categorized as having chronic disease if they reported one or more of these.

The screening question for MSCs was: “Have you during the last year suffered from pain and/or stiffness in muscles and joints that have lasted continuously for at least 3 months?” (yes/no). At the medical examination, participants who answered “yes” to this question were asked to indicate which of the following body regions were affected: neck/shoulder, arm, upper back, lumbar back, chest/stomach, and hip/leg. Participants were categorized as having widespread MSCs if they reported pain and/or stiffness for at least 3 months during the past year in all of the following regions: the axial skeleton (neck/shoulder, upper back, or lumbar back), above the waist (neck/shoulder, arm, upper back, or chest/stomach), and below the waist (lumbar back or hip/leg) [31]. Weight and height were also measured during the medical examination, with participants wearing light clothing and without shoes, and these measurements were used to calculate body mass index (BMI).

The outcome variables were all-cause mortality and cause-specific mortality. Information on date of death and emigration date was collected via record linkage to the National Register of Norway using the national personal identification number. Information on cause of death was collected from the National Cause of Death Registry also using the national personal identification number, and recorded according to the International Classification of Diseases, 9^th^ Revision (ICD-9) until 31 December 1995 and according to the International Classification of Diseases, 10^th^ Revision (ICD-10) thereafter. Causes of death were categorized as “cardiovascular disease death” (ICD-9 codes: 390–459; ICD-10 codes: I00-99) or “cancer death” (ICD-9 codes: 140–239, and ICD-10 codes: C00-97). Information on all-cause mortality was updated until 17 October 2015 and cause-specific mortality was updated until 31 December 2012. After the linkage processes, participants were made anonymous.

### Statistics

**Fig 1:**
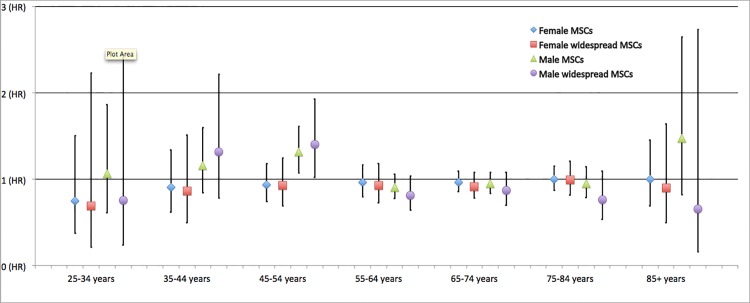
Hazard ratios (HRs) and 95% confidence intervals (CIs) stratified by sex and 10-year age groups from a univariable Cox regression model of all-cause mortality among participants with musculoskeletal complaints (MSCs) or widespread MSCs in a prospective cohort study of an adult general population in Northern Norway. The Tromsø study.

Participants were followed from the time of entry into Tromsø 4 until death, date of emigration, or the end of follow-up (17 October 2015). Descriptive statistics were performed using the chi-square test for categorical variables and the independent-samples t-test for continuous variables. Cox regression analyses were used to determine the risk estimates of death expressed as hazard ratios (HR) with 95% confidence intervals (CI). The analyses were first performed unadjusted, then adjusted for age, and finally a multivariable Cox regression model was performed. The Cox regression analyses were performed on those with MSCs vs those with no MSCs for all-cause death, cardiovascular death, and cancer death. Analyses on widespread MSCs vs no MSCs were undertaken in the same way. Additionally, a univariable Cox regression model on the presence of MSCs was stratified by 10-year age groups and graphically presented (Fig 1).

The statistical analyses were performed in SPSS version 21. All tests were performed two-sided and a p-value <0.05 was considered significant.

### Ethics

The Regional Committee of Research Ethics and the Norwegian Data Inspectorate approved the Tromsø 4 survey, and the approval covered linkage to the National Register of Norway and the National Cause of Death Registry for collection of mortality data. Written informed consent was obtained from all participants.

## Results

### Sample characteristics

Of the 26,977 participants included in this study, 9639 (35.7%) reported MSCs at baseline and 3445 (12.8%) fulfilled the criteria of widespread MSCs. By the end of follow-up in October 2015, a total of 375 participants had emigrated and 5693 (21.1% of the included participants, 2794 women and 2905 men) had died. The mean time and standard deviation from entry into the study until death or censoring was 18.6 ± 4.87 years for the all-cause mortality analyses, and 16.4 ± 3.93 for cause-specific analyses, with an overall of 502,040.3 and 442,045.8 person-years of follow-up, respectively.

Compared to those without MSCs at baseline, participants reporting MSCs were significantly older (49.96 vs 45.19 years) and had a slightly higher BMI (25.60 vs 24.95). They were also more likely to be current smokers (39.9% vs 35.1%), have mental health complaints (11.3% vs 4.8%), a lower educational level (78.0% vs 66.1%), a sedentary physical activity level (12.2% vs 8.0%), and 60.7% were women compared to 48.0% of those without MSCs (all p-values <0.001). The prevalence of self-reported chronic diseases was significantly higher among those with MSCs at baseline compared to those without MSCs (20.3% vs 14.3%). The descriptive characteristics were all significantly different when comparing the category of widespread MSCs and no MSCs (all p-values <0.001) (Table 1).

**Table 1:** Baseline characteristics and descriptive statistics of the study sample by category of musculoskeletal complaint (MSC) in a prospective cohort study of an adult general population in Northern Norway. The Tromsø Study.

Characteristics	No MSCs (n = 17,309)	MSCs (n = 9639)	Widespread MSCs (n = 3445)
Age in years, mean (SD)	45.19 (15.0)	49.96 (14.99)	50.48 (13.9)
Female sex, n (%)	8312 (48.0)	5850 (60.7)	2383 (69.2)
Current smoker, n (%)	6083 (35.1)	3843 (39.9)	1481 (43.0)
CONOR-MHI ≥2.15**, n (%)	803 (4.8)	1050 (11.3)	462 (14.0)
<13 years of education, n (%)	11,410 (66.1)	7491 (78.0)	2805 (81.8)
Body mass index, mean (SD)	24.95 (3.7)	25.60 (4.13)	25.74 (4.27)
Sedentary physical activity level, n (%)	1364 (8.0)	1167 (12.2)	452 (13.3)
Chronic disease, n (%)	2467 (14.3)	1958 (20.3)	754 (21.9)

Chi-square test for categorical and independent samples t-test for continuous variables (MSCs vs No MSCs and Widespread MSCs vs No MSCs); All p-values <0,001.

**MHI = mental health index.

### Risk of mortality

In the crude Cox regression model, both sexes had increased risk of death from all causes; women with MSCs at baseline (HR = 1.36, 95% CI: 1.26–1.46) and with widespread MSCs (HR = 1.32, 95% CI, 1.19–1.45); corresponding figures for men were HR = 1.35 (95% CI: 1.25–1.45) and HR = 1.26 (95% CI: 1.11–1.43). The risk of cardiovascular disease death and cancer death was higher among those with MSCs and widespread MSCs in both sexes. Furthermore, the age-stratified univariable Cox regression revealed consistent HRs in all of the 10-year age groups (Fig 1).

However, after adjusting for age in the Cox regression analyses, women with MSCs (HR = 0.98, 95% CI: 0.91–1.06) and widespread MSCs (HR = 0.96, 95% CI: 0.87–1.06) did not have any increased risk of all-cause mortality. The same was true for men (HR = 1.01, 95% CI: 0.93–1.08 for MSCs; HR = 0.95, 95% CI: 0.84–1.08 for widespread MSCs). The risk of death from cardiovascular disease and cancer did not increase for women or men in the age-adjusted regression models (Table 2).

**Table 2:** Number of deaths (n) and hazard ratios (HRs) and 95% confidence intervals (CIs) from univariable, age-adjusted and multivariable Cox regression models of all-cause and cause-specific mortality among participants with musculoskeletal complaints (MSCs) or widespread MSCs in a prospective cohort study of an adult general population in Northern Norway. The Tromsø Study.

		Female	Male	Female	Male	Female	Male
Outcome	N of deaths (%)	Crude HR (95% CI)	Crude HR (95% CI)	Age-adjusted HR (95% CI)	Age-adjusted HR (95% CI)	Multivariable^a^ HR (95% CI)	Multivariable^a^ HR (95% CI)
**All-cause mortality**							
- MSCs	2376 (24.6)	**1.36 (1.26–1.46)**	**1.35 (1.25–1.45)**	0.98 (0.91–1.06)	1.01 (0.93–1.08)	0.93 (0.85–1.01)	0.93 (0.85–1.01)
- Widespread MSCs	806 (23.4)	**1.32 (1.19–1.45)**	**1.26 (1.11–1.43)**	0.96 (0.87–1.06)	0.95 (0.84–1.08)	0.90 (0.80–1.01)	0.87 (0.76–1.00)
**CVD mortality**							
- MSCs	743 (7.7)	**1.41 (1.23–1.61)**	**1.41 (1.23–1.62)**	1.05 (0.92–1.20)	1.06 (0.93–1.22)	0.94 (0.80–1.10)	0.98 (0.85–1.14)
-Widespread MSCs	252 (7.3)	**1.31 (1.10–1.58)**	**1.42 (1.14–1.77)**	1.04 (0.86–1.24)	1.12 (0.90–1.40)	0.92 (0.73–1.14)	1.07 (0.84–1.35)
**Cancer mortality**							
- MSCs	578 (6.0)	**1.17 (1.01–1.36)**	**1.30 (1.12–1.51)**	0.88 (0.76–1.03)	0.98 (0.85–1.14)	0.91 (0.77–1.08)	0.94 (0.80–1.11)
-Widespread MSCs	218 (6.3)	**1.26 (1.04–1.53)**	**1.39 (1.10–1.76)**	0.94 (0.77–1.14)	1.07 (0.84–1.35)	0.95 (0.76–1.20)	1.00 (0.77–1.30)

Bold text = significant results. CVD = cardiovascular disease.

^**a**^Adjusted for age, smoking, mental health complaints, educational level, body mass index, leisure time physical activity, self-reported chronic diseases (cancer, CVD, diabetes or asthma).

The results from the age-adjusted analyses were not altered in the multivariable Cox regression models–the risk of death from all causes among women with MSCs (HR = 0.93, 95% CI: 0.85–1.01) and widespread MSCs (HR = 0.90, 95% CI: 0.80–1.01) did not increase. Among men, the multivariable regression model showed no significant associations between all-cause mortality and MSCs (HR = 0.93, 95% CI: 0.85–1.01) or widespread MSCs (HR = 0.87, 95% CI: 0.76–1.00). The analyses on cause-specific death did not produce additional information.

## Discussion

This large prospective cohort study of an adult population from the Arctic region of Northern Norway examined the relationship between self-reported pain and/or stiffness in muscles or joints (musculoskeletal complaints: MSCs) and mortality. The crude analyses revealed a 35% to 36% increased risk of death in women and men with self-reported MSCs. The corresponding figures for the more serious cases (widespread MSCs) were lower in both women and men. However, the significantly increased mortality risk found in the crude analyses was lost in the age-adjusted Cox regression models, which could not be explained by systematic changes in mortality risk by increasing age (Fig 1). The results did not change significantly in the multivariable Cox regression models. In sum, this indicates that if there is a true increase in the risk of death among our participants reporting MSCs, it must be attributed to other negative health determinants. Our study highly suggests that presence of MSCs are not independent predictors of mortality. These findings are supported by other authors [13–17], including a Norwegian cohort study published in 2016 by Åsberg et al [19].

We previously demonstrated that the prevalence of severe MSCs increased up to the age of 50–59 years, and decreased thereafter [3]. In light of our current findings, it is unlikely that premature death can explain the declining prevalence of severe MSCs after the age of 60. It may instead reflect an actual decrease in the burden of MSCs in some parts of the population, which could be interesting to explore further from a therapeutic point of view.

To our knowledge, only a few studies found increased mortality related to MSCs after adjusting for potential confounders [10, 32, 12, 11]; most of them had smaller cohorts and a shorter follow-up time compared to the present study. The latest systematic review in this field stressed the importance of using a harmonized methodology for future robust determination of whether chronic pain increases the risk of mortality and for identification of possible pathways [26]. Even though the study by Åsberg et al study was carried out in a more southern part of Norway with a more rural population, there are several methodological similarities between these two studies. Both were carried out in large general populations of Norwegian women and men with good participation rates, and the variables on MSCs and mortality were highly comparable. In addition, Norway has a nationwide social welfare system; hence, both populations experience similar level of social support. In summary, the present study, together with the Åsberg et al study, addresses the concerns raised by Smith et al in their systematic review, and we claim that the results of these studies together robustly determine that MSCs alone do not contribute to an increased risk of death. However, individuals reporting MSCs might have poorer health than those without MSCs due to coexisting conditions, and this should be reviewed carefully by health workers and researchers when dealing with individuals suffering from MSCs. From the patients’ perspective these findings might also be interesting to review. Even though MSCs can influence on the individuals working capacity and quality of life, focusing on reduction of negative lifestyle factors are still more important than MSCs in preventing premature death.

### Strengths and limitations

There are several methodological considerations that should be carefully reviewed in the present study. The follow-up time of up to 21 years is one of the major strengths. If we assume that the exposure time of MSCs needs to be several months or years before it influences mortality rates, our study design should reveal any such associations. However, we only have information about MSCs at baseline and that they lasted for at least 3 months during the past year. Thus, it is possible that some of our participants who reported MSCs at baseline no longer had them after the baseline examination or during follow-up. On the other hand, some participants might have experienced MSCs during follow-up, which might result in underestimation of the associated risk of death.

To a large extent, we based our analyses on self-reported health information, which in itself might produce bias. The information on cancer was taken from the second questionnaire (Q2), which was completed at the time of the medical examination. However, Q2 had a slightly lower response rate than did the first questionnaire (Q1), and this contributed to the high number of missing values for the self-reported disease variable. However, we conducted a sensitivity analysis including only those respondents that answered Q2 (N = 22,981) and the results did not differ from those presented in Table 2.

The screening question on MSCs has been found to have acceptable reliability [5]. Furthermore, a validation study of self-reported stroke data from the same study population has been performed, and the authors concluded that stroke prevalence could be assessed through questionnaire information in epidemiological research [33]. If the same applies for other diseases, we can assume that our self-reported data on chronic diseases is credible. The variables on mental health complaints (CONOR-mental health index) have been validated and found to correspond well with the widely used Hospital Anxiety and Depression Scale (HADS) and Hopkins Symptom Check List (HSCL-10) [29]. Regarding the validity of mortality data, all deaths of Norwegian inhabitants are reported to the National Register of Norway through a standardized death certificate completed by a medical doctor who also determines the cause of death. As such, there might be some uncertainty connected to the cause of death since autopsy is not obligatory in Norway. However, the date of death must be regarded as highly valid, which applies to our all-cause mortality analysis.

## Conclusion

The results of this study do not support that MSCs are an independent risk factor of mortality. Increased rates of death among individuals with self-reported MSCs could be attributed to other coexisting characteristics and risk factors, and reduction of negative lifestyle factors are still more important than MSCs in preventing premature death.
